# The First Medical Research Conference in Hama, Syria: Students' Experience to Encourage and Improve the Research Reality during the War

**DOI:** 10.1055/s-0044-1801258

**Published:** 2025-01-10

**Authors:** Kenana Tawashi, Maher Almousa, Yamama Tawashi, Ahmed Al-Beiruti, Ahmad Khaled AlFaris

**Affiliations:** 1 Department of Oncology, Faculty of Medicine, Hama University, Hama, Syria; 2 Department of Internal Medicine, Faculty of Medicine, Hama University, Hama, Syria

Syria has been affected by a devastating war for the past 14 years; the people are suffering to get their basic rights in all aspects of life. In 2015, four out of every five persons here lived below the national poverty line, with two-thirds of the population suffering from severe poverty, and the condition has been extremely deteriorating since that time. 1 2 One of the most affected areas of life is education. While people are trying to meet their food and nonfood basic needs, it is hard to maintain their education level and get an appropriate school and university life, especially with internal and external migration, using schools as shelters, lack of the sources and equipment, power blackouts, and students working to support themselves and their families. In this harsh situation, our university (Hama University) was established in 2014, including the College of Medicine and many other colleges. Therefore, the students here study in a newly established university in wartime, which doubles their struggles. Scientific research and its skills are among the most neglected components despite their importance in increasing scientific curiosity and enhancing critical thinking, problem-solving skills, and clinical judgment. Students involved in research during their university lives are more productive after graduation, maybe because they feel that they contribute to their field and strengthen their professional identity. 3 4 During our fourth year in college, we developed a keen interest in scientific research after reading about other students' experiences in other universities like Damascus and Aleppo Universities. We sought guidance from senior colleagues, online courses on platforms like YouTube and Coursera, and books. After 1 year of learning and trying, we published our first case reports. 5 6 7 Case reports played a crucial step, not only because they described unusual findings but also because they were an appropriate first step for students in our condition to learn searching in specific topics, academic writing, references management, and paper submission, which formed the basic infrastructure for more research. 4 Students in the Middle East faced many challenges during their work in medical research, including insufficient training and opportunities, lack of resources and equipment, inadequate funding, limitations in time due to the large medical curriculum and long clinical training hours, shortage of faculty members who are interested in medical research and have sufficient time to supervise research, and lack of support and stimulation for both mentors and students. 3 In addition to the mentioned challenges that students in developing countries face, there are many other unique challenges like electricity and Internet blackouts, difficulty in navigating research processes, and inaccessibility to scientific journals for undergraduate medical students. All these challenges encouraged us to take a step that will pave the way for other students and shorten their time and effort. So, we thought of a way to spread the culture of medical research to the greatest possible number of students, residents, and specialists in our region, and concluded that organizing a conference would be the best choice. Conferences form an important platform for highlighting the new trends and directions in a specific subject, opening the door to discussions about controversial topics, holding the key persons who are professionals and interested in the field, and offering opportunities for jobs and networking with people from other regions. 8 In our case, organizing a conference was a big challenge, especially for students in medical schools who did not have any previous experience in the organization for such a big event. There were many aspects that needed to be taken care of like funding, organization, sponsors, guests' choice, topics for discussion, and target audience. First, we connected with the Syrian Society for Social Development and United Nations High Commissioner for Refugees (UNHCR) and presented our proposal; they accepted and admired it, as they had not funded such a project before. In addition, we connected with the Doctors Syndicate in Hama to sponsor our conference. With their support, we started organizing our conference. We chose a location that could host the largest number of interested people and we connected with key persons in medical research in the Arab world and Syria. After consulting with these experts, we identified topics that would help students to start their journey in the medical research from zero, and we decided to target students, residents, and specialists in this conference as they all complement each other's works. It took more than 6 months to prepare for this big and unprecedented event in the best possible way. The conference was held in Hama city and more than 450 students, residents, and physician doctors attended the conference (while we expected <200 to attend). It lasted 2 days and included many important and variant topics like the history of medical research, types of scientific studies, structure of scientific papers, research question, datasets, choosing journals, and paper submission. Special topics were also discussed like artificial intelligence and its applications in research and statistical concepts that help researchers to understand and analyze data. The conference was followed by five training courses that were held in the Doctors Syndicate; we trained 20 students, residents, and specialists in each course in scientific research and medical statistics. These courses were more dedicated and included interactive discussions and practical activities. The feedback, which we got from the attendants of the conference and courses, was amazing and unforeseen. The sponsors admired the high quality of the organization and the importance of the discussed topics, and they expressed their intention to support such programs and projects in the future and share this unique and productive experience with other organizations to support the education and research landscape in developing war-torn countries and highlight the short-term and long-term impacts on the individual and the society. In addition, beginners in medical research expressed their appreciation for the simplicity, appeal, and importance of the information and discussions. We believe that our experience represents a milestone to inspire others who are interested in medical research to share their knowledge and expertise with others and to encourage those who are just beginning their journey showing that even in the face of harsh conditions that we live in, we can make a difference and conduct our research.
